# Tumor-immune profiling of CT-26 and Colon 26 syngeneic mouse models reveals mechanism of anti-PD-1 response

**DOI:** 10.1186/s12885-021-08974-3

**Published:** 2021-11-13

**Authors:** Yosuke Sato, Yu Fu, Hong Liu, Min Young Lee, Michael H. Shaw

**Affiliations:** 1 Immuno-oncology Drug Discovery Unit, Millennium Pharmaceuticals, Inc. a wholly owned subsidiary of Takeda Pharmaceutical Company Limited, 40 Landsdowne St, Cambridge, MA 02139 USA; 2 Guardant Health, 720 3rd Ave Suite 2100, Seattle, WA 98104 USA; 3 Checkmate Pharmaceuticals, 245 Main St, Cambridge, MA 02142 USA

**Keywords:** Immune checkpoint blockade, Anti-PD-1, Syngeneic model, CT-26, Colon 26, Anti-tumor activity, Wnt

## Abstract

**Background:**

Immune checkpoint blockade (ICB) therapies have changed the paradigm of cancer therapies. However, anti-tumor response of the ICB is insufficient for many patients and limited to specific tumor types. Despite many preclinical and clinical studies to understand the mechanism of anti-tumor efficacy of ICB, the mechanism is not completely understood. Harnessing preclinical tumor models is one way to understand the mechanism of treatment response.

**Methods:**

In order to delineate the mechanisms of anti-tumor activity of ICB in preclinical syngeneic tumor models, we selected two syngeneic murine colorectal cancer models based on in vivo screening for sensitivity with anti-PD-1 therapy. We performed tumor-immune profiling of the two models to identify the potential mechanism for anti-PD-1 response.

**Results:**

We performed in vivo screening for anti-PD-1 therapy across 23 syngeneic tumor models and found that CT-26 and Colon 26, which are murine colorectal carcinoma derived from BALB/c mice, showed different sensitivity to anti-PD-1. CT-26 tumor mice were more sensitive to the anti-PD-1 antibody than Colon 26, while both models show similarly sensitivity to anti-CTLA4 antibody. Immune-profiling showed that CT-26 tumor tissue was infiltrated with more immune cells than Colon 26. Genomic/transcriptomic analyses highlighted thatWnt pathway was one of the potential differences between CT-26 and Colon 26, showing Wnt activity was higher in Colon 26 than CT-26. .

**Conclusions:**

CT-26 and Colon 26 syngeneic tumor models showed different sensitivity to anti-PD-1 therapy, although both tumor cells are murine colorectal carcinoma cell lines from BALB/c strain. By characterizing the mouse cells lines and tumor-immune context in the tumor tissues with comprehensive analysis approaches, we found that CT-26 showed “hot tumor” profile with more infiltrated immune cells than Colon 26. Further pathway analyses enable us to propose a hypothesis that Wnt pathway could be one of the major factors to differentiate CT-26 from Colon 26 model and link to anti-PD-1 response. Our approach to focus on preclinical tumor models with similar genetic background but different sensitivity to anti-PD-1 therapy would contribute to illustrating the potential mechanism of anti-PD-1 response and to generating a novel concept to synergize current anti-PD-1 therapies for cancer patients.

**Supplementary Information:**

The online version contains supplementary material available at 10.1186/s12885-021-08974-3.

## Background

Cancer immunotherapy, with the recent focus on immune checkpoint blockades (ICB), is a revolutionary approach and has shown great clinical benefit in multiple tumor types with an intent to “cure.” ICB therapies, such as anti-cytotoxic T lymphocyte-associated antigen 4 (CTLA-4) and anti-programmed cell death protein 1 (PD-1) antibodies, have clinical response rates in the range 10–35% as single agents [1], and the anti-tumor response is not sufficient in many patients and responses are more common in specific tumor types. Moreover, mechanisms of anti-tumor efficacy of the ICB in the responders are not completely understood. Understanding how anti-CTLA-4 and anti-PD-1 checkpoint blockade therapies work will be critical for effectively combining them with other anti-cancer therapeutic approaches such as immune-, radio-, chemo- and targeted therapy to improve overall response rate [2].

For clarifying how ICB work and induce anti-tumor effect in the cancer patients, it is critical to understand contributions of both host immune cells and cancer cells, which are key components of the tumor-micro-environment (TME) and are potential target cells to ICB responsiveness. Based on tumor-type, density and location of immune cells within the tumor site, there are four proposed types of tumors: hot, excluded, immunosuppressed and cold tumors [1]. While hot/inflamed tumors have the greatest potential to respond to ICB, those lacking tumor infiltrated T cells are often resistant to ICB [1]. Multiple studies have found a positive correlation between tumoral PD-L1 expression and anti-PD-1 response or overall survival in some tumor types [3]. These evidence suggest that presence of target cells and expression of target molecules of ICB in tumors are the key factors to determine treatment response.

Genomic/transcriptomic analyses of tumor tissues between ICB responder and non-responders reveal that there are differences of genomic and transcriptional profile in the tumor [1, 4]. The number of non-synonymous single nucleotide variants (nsSNVs) in a tumor, referred as the tumor mutation burden (TMB), is thought to increase the generation of immunogenic peptides and hence influence ICB response in patients [3]. Indeed, significant association between high TMB and ICB response have been reported in a variety of tumor types [5] including urothelial carcinoma [6], small cell lung cancer [7], independent cohort of non-small cell lung cancer (NSCLC) [8–10], and melanoma [11–13]. Genetic alteration of the tumors contributed to immunosuppressive mechanisms occurred in the TME, which hindered not only the natural hot immune responses but also the efficacy of cancer immunotherapies [1]. The tumor-intrinsic immunosuppression usually involves the activation of various oncogenic pathways, including Wnt–β-catenin [14, 15], mitogen-activated protein kinase (MAPK) [16, 17], Janus kinase (JAK)–signal transducer and activator of transcription 3 (STAT3) [18] and nuclear factor-κB (NF-κB) signaling pathways [19]. The engagement of these pathways results in the expression of cytokines and chemokines that mediate the exclusion of T cells from the TME, or, alternatively, the repression of factors that facilitate T cell recruitment and therefore affecting response to ICB. More work is needed to delineate the response/resistance mechanism to current ICB therapy [20]. Host-derived immune cells and tumor cells are known to interact with each other and have significant impact on anti-tumor efficacy of ICB treatment. The use of preclinical immunocompetent tumor models, where immune system is working properly, is one way to understand the mechanism of ICB response that allows to analyze both tumor cells and host immune cells in parallel.

In this paper, we first screened 23 different syngeneic tumor models treated with anti-PD-1 and found that two colorectal carcinoma CT-26 and Colon 26 tumor models shared similar genetic background but exhibited different sensitivity to anti-PD-1 therapies. Next, we characterized both models in-depth by immune-profiling and genomic/transcriptomic analyses. Our data and analyses proposed a hypothesis that Wnt pathway is potentially one of the major components contributing to the different sensitivity to anti-PD1 therapy in CT-26 and Colon 26 models.

## Methods

### Animals

BALB/c, C57BL/6 and DBA/2 mice were purchased from Beijing Vital River Laboratory Animal Technology Co., Ltd. (Beijing, China) and from Shanghai Lingchang Laboratory Animal Co. All animal studies were performed under Takeda and WuXi’s IACUC policies, and AAALAC’s ethical approval and regulations.

### Mouse cell lines

4 T1, A20, B16F10, C1498, CT-26, E.G7-OVA, EL4, EMT-6, J558, JC, KLN205, L1210, L5178-R, LLC1, P388D1, RENCA and WEHI-3 were purchased from ATCC (Manassas, VA, USA). Colon 26 was purchased from RIKEN BioResource Research Center (Tsukuba, Ibaraki, Japan) and Nanjing CoBioer Biotechnology Co., Ltd. (Nanjing, China). H22 was purchased from China Center for Type Culture Collection (Beijing, China). LLC1-Luc was purchased from PerkinElmer (Waltham, MA, USA). MC38 and Panc02 were purchased from Obio Technology (Shanghai) Corp., Ltd. (Shanghai, China). RM-1 was purchased from Cell Bank of Shanghai Institutes for Biological Sciences of the Chinese Academy of Sciences (Shanghai, China). All cells were culture by following instructions of the providers.

### Antibodies

Anti-mouse CTLA-4 (CD152) mAb 9D9 and anti-mouse PD-1 (CD279) mAb RMP1–14 were purchased from Bio-X Cell Inc. (West Lebanon, NH, USA).

### Murine tumor models

To establish murine breast cancer model 4 T1, 4 × 10^5^ cells were subcutaneously implanted into the right flank of female BALB/c mice. To establish murine breast cancer model EMT6 and JC, 2 × 10^5^ cells were subcutaneously implanted into the right flank of female BALB/c mice. To establish murine colon adenocarcinoma cancer model Colon26 and CT-26, 3 × 10^5^ cells were subcutaneously implanted into the right flank of female BALB/c mice. To establish murine hepatoma cancer model H22, 4 × 10^5^ cells were subcutaneously implanted into the right flank of female BALB/c mice. To establish murine kidney carcinoma cancer model RENCA, 2 × 10^5^ cells were subcutaneously implanted into the right flank of female BALB/c mice. To establish murine leukemia cancer model WEHI-3, 1 × 10^5^ cells were subcutaneously implanted into the right flank of female BALB/c mice. To establish murine diffuse large B cell lymphoma cancer model A20, 4 × 10^5^ cells were subcutaneously implanted into the right flank of female BALB/c mice. To establish murine plasmacytoma cancer model J558, 1 × 10^5^ cells were subcutaneously implanted into the right flank of female BALB/c mice. To establish murine leukemia cancer model C1498, 4 × 10^5^ cells were subcutaneously implanted into the right flank of female C57BL/6 mice. To establish murine colon adenocarcinoma cancer model MC38, 1 × 10^6^ cells were subcutaneously implanted into the right flank of female C57BL/6 mice. To establish murine Lewis lung carcinoma cancer model LLC1 and LLC1-Luc, 1 × 10^5^ cells were subcutaneously implanted into the right flank of female C57BL/6 mice. To establish murine lymphoma cancer model E.G7-OVA, 1 × 10^5^ cells were subcutaneously implanted into the right flank of female C57BL/6 mice. To establish murine lymphoma cancer model EL4, 0.8 × 10^5^ cells were subcutaneously implanted into the right flank of female C57BL/6 mice. To establish murine melanoma cancer model B16F10, 0.8 × 10^5^ cells were subcutaneously implanted into the right flank of female C57BL/6 mice. To establish murine pancreatic ductal adenocarcinoma model Panc02, 1 × 10^6^ cells were subcutaneously implanted into the right flank of female C57BL/6 mice. To establish murine prostate carcinoma model RM-1, 8 × 10^5^ cells were subcutaneously implanted into the right flank of female C57BL/6 mice. To establish murine leukemia model L1210, 0.2 × 10^5^ cells were subcutaneously implanted into the right flank of female DBA/2 mice. To establish murine lung carcinoma model KLN205, 4 × 10^5^ cells were subcutaneously implanted into the right flank of female DBA/2 mice. To establish murine lymphoma model L5178-R and P388D1, 2 × 10^5^ cells were subcutaneously implanted into the right flank of female DBA/2 mice. Tumor growth was monitored with vernier calipers, and the mean tumor volume was calculated using the formula (0.5 ´ [length ´ width2]).

When the mean tumor volume reached approximately 60 mm^3^, animals were randomized into treatment groups (*n* = 10/group in all efficacy studies) and dosing was initiated on Day 0 of the study. Tumor size and body weight were measured three times weekly. During the observation period, animals bearing oversized tumor exceeding 2000 mm^3^ were sacrificed. Anti-mouse CTLA-4 (10 mg/kg) and anti-mouse PD-1 (10 mg/kg) were administered intraperitoneally on day 0, 3, 7, 10, 14 and 17.

### Flow cytometry

BALB/c mice were inoculated with 3 × 10^5^ CT-26 or Colon 26 cells. When the tumor volume reached approximately 100 mm^3^ at day 12 (CT-26) or at day 11 (Colon 26) after the inoculation, tumor tissues were harvested (*n* = 4). To prepare tissues for flow cytometry, tumor samples were digested by using a tumor dissociation kit (MACS). Spleens and draining lymph nodes were mechanically disrupted into single-cell suspensions through a 70 μm cell strainer. Red blood cells were lysed in Red Blood Cell Lysis Solution (BD Bioscience). Cells were washed, pelleted and re-suspended in D-PBS solution (Corning). For each sample, 1 × 10^6^ cells were treated with anti-mouse CD16/CD32 (BD Pharmingen) and then stained with a defined panel containing live/dead stain and with various labeling antibodies. All antibodies were purchased from BD Pharmingen, BioLegend, or eBioscience as indicated. Live/Dead cell stain kit was purchased from Invitrogen. Data were measured on BD LSR Fortessa and analyzed by using FlowJo software. Flow cytometry antibodies used in this study were purchased from BD Pharmingen (anti-CD8 [53–6.7], anti-CD4 [GK1.5], anti-CD19 [1D3], anti-CD25 [PC61], anti-CD335 [29A1.4], anti-CD45 [30-F11], anti-CD3 [17A2], anti-Ly6G [1A8], anti-MHCII [M5/114.15.2], anti-CD11c [HL3], anti-Ly6C [AL-21], anti-CD206 [MR5D3]), BioLegend (anti-PD-1 [29F.1A12], anti-CD11b [M1/70], anti-CTLA-4 [UC10-4B9], anti-CD44 [IM7], anti-CD62L [MEL-14], anti-F4/80 [BM8]) or eBioscience (anti-FoxP3 [FJK-16 s]).

### Data analysis

Flow cytometry data were analyzed with FlowJo. Statistical analyses were done with Prism 8 (GraphPad Software). Kaplan-Meier curves were analyzed with the log-rank test. Student’s t test was used for comparison of immune cells between CT-26 and Colon 26 tumor models.

### RNA-seq

Cell line and tumor tissue RNA was purified using RNeasy MinElute Cleanup Kit. RNA quality was assessed using RNA 6000 Nano Reagents. cDNA RNA library was constructed using TruSeq mRNA Sample Preparation Kit (illumina). The first step in the workflow involved purifying the poly-A containing mRNA molecules using oligo-dT attached magnetic beads. Following purification, the mRNA was fragmented into small pieces using divalent cations under elevated temperature. The cleaved RNA fragments were copied into first strand cDNA using reverse transcriptase and random primers. Second strand cDNA synthesis followed, using DNA PolymeraseI and RNase H. The cDNA fragments then went through an end repair process, the addition of a single ‘A’ base, and then ligation of the adapters. The products were then purified and enriched with PCR to create the final cDNA library. The clusters of the cDNA library were generated on Illumina cBOT cluster generation system with HiSeq X HD PE Cluster Kits (illumina). Paired-end sequencing was performed using an Illumina HiSeq X following Illumina-provided protocols for 2 × 150 paired-end sequencing.

### Whole exome sequencing

Cell line and tissue DNA was purified using QIAamp DNA Mini Kit. DNA quality was assessed using Qubit dsDNA HD Assay Kit. DNA library was constructed using SureSelect XT library prep kit (Agilent). The first step in the workflow involved DNA fragment, constructing libraries and amplifying the libraries. After that, the library was hybridized using SureSelect XT reagent and protocol and the captured libraries were obtained. The products were then purified and enriched with PCR to create SureSelect-enriched indexed NGS samples. The clusters of the captured library were generated on Illumina cBOT cluster generation system with HiSeq X HD PE Cluster Kits (illumina). Paired-end sequencing was performed using an Illumina HiSeq X following Illumina-provided protocols for 2 × 150 paired-end sequencing.

### Bioinformatics

To quantify transcript abundance, we calculated the raw count for each gene from RNA-seq data using “Report gene/transcript counts” in Array Studio (version 10.0.1.118) and Ensembl.R78 annotation. Then DESeq2 (version 1.24.0) with default parameters was used to determine differentially expressed genes, requiring adjusted *p*-value ≤0.1 and the absolute log2 fold change ≥1. Ingenuity Pathway Analysis was used to determine the significantly enriched pathways.

Tumor mutational burden was calculated by dividing the number of non-synonymous mutations called using exome sequencing data by the length of mouse CDS regions. SNPs were called and annotated using “summarize variant data” and “annotate variant table report,” respectively. We compared CT-26 and Colon 26 tumors at each size (100, 400 and 800 mm3) separately to obtain 3 sets of differentially expressed genes, and then merged these gene sets to obtain the differentially expressed genes between CT-26 and Colon 26 tumors. For clustering analysis of Wnt ligand and Wnt-regulated gene expression, pheatmap function of the pheatmap R package and agglomerative hierarchical clustering with ward.D2 linkage method were used. The gene expression levels (fpkm, fragments per kilobase of transcript per million mapped reads) plus 1 were log2-transformed (log2(fpkm+ 1)), and then z-transformed before clustering.

## Results

### CT-26 tumor-bearing mice are more sensitive to anti-PD-1 checkpoint blockade than Colon 26 tumor-bearing mice, while both tumor models are sensitive to anti-CTLA-4 blockade

**Fig. 1:**
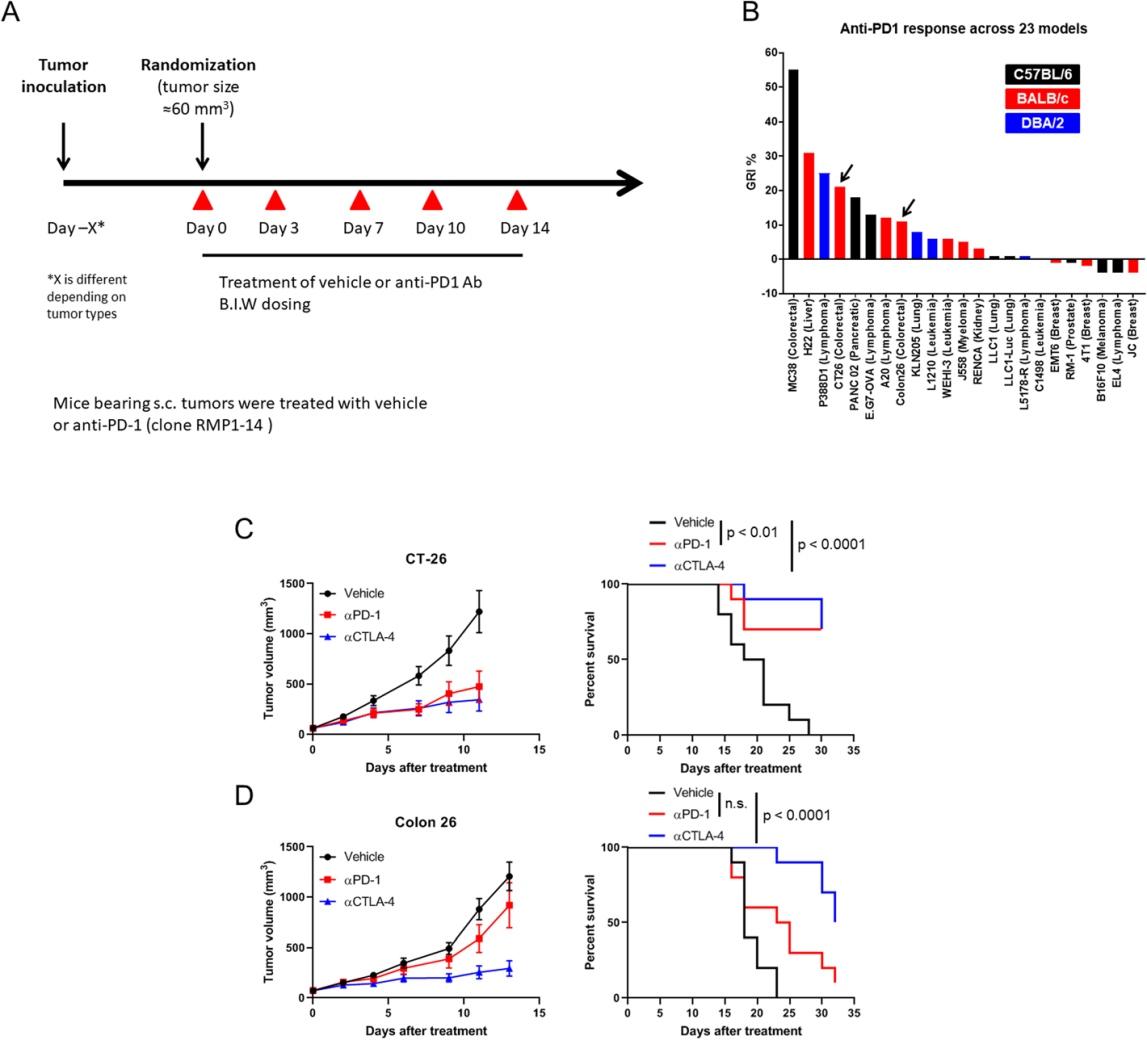
CT-26 tumor mice are more sensitive to anti-PD-1 checkpoint blockade than Colon 26 tumor mice. (**A**) A scheme of in vivo studies for anti-PD-1 antibody across 23 syngeneic tumor models. (**B**) Growth rate inhibition (%) of anti-mouse PD-1 across 23 syngeneic tumor models of different background mouse strains. (**C**) Growth and survival of BALB/c mice bearing CT-26 tumors treated with vehicle (PBS), anti-mouse PD-1 and anti-mouse CTLA-4. When the mean tumor volume reached approximately 60 mm^3^, animals were randomized into treatment groups (*n* = 10/group) and dosing was initiated on Day 0 of the study. (**D**) Growth and survival of BALB/c mice bearing Colon 26 tumors treated with vehicle (PBS), anti-mouse PD-1 and anti-mouse CTLA-4. When the mean tumor volume reached approximately 60 mm^3^, animals were randomized into treatment groups (*n* = 10/group) and dosing was initiated on Day 0 of the study. (Left) Tumor size as measured by vernier calipers, and the data shown in all panels are the mean (*n* = 10/group) ± SEM. (Right) Survival curve comparing treatment groups. Mice were euthanized when tumors reached 2000 mm^3^. n.s., nonsignificant

To investigate the sensitivity of various preclinical tumor models to immune checkpoint inhibitors, anti-PD-1 monoclonal antibody (mAb) was tested across 23 different syngeneic tumor mouse models on C57BL/6, BALB/c and DBA/2 genetic background mice (Fig. 1A). We found that anti-PD-1 treatment induced higher growth rate inhibition (GRI) in CT-26 tumor bearing mice than Colon 26, although both tumors are murine colorectal carcinoma derived from BALB/c mice (Fig. 1B). Next, to investigate whether the different response between CT-26 and Colon 26 was specific for anti-PD-1 or common for ICB, we tested anti-PD-1 and anti-CTLA-4 mAb in both CT-26 and Colon 26 tumor-bearing mice. Consistent with the previous anti-PD-1 screening, anti-PD-1 treatment induced significant tumor growth inhibition and significantly promoted survival in CT-26 tumor-bearing mice, but not in Colon 26 tumor-bearing animals (Fig. 1C and D, Additional file: Fig. S1). On the other hand, anti-CTLA-4 treatment induced significant tumor growth inhibition and significantly promoted survival in both models (Fig. 1C and D, Additional file: Fig. S1).

### CT-26 tumors are infiltrated with more anti-tumor immune cells than Colon 26 tumors

**Fig. 2:**
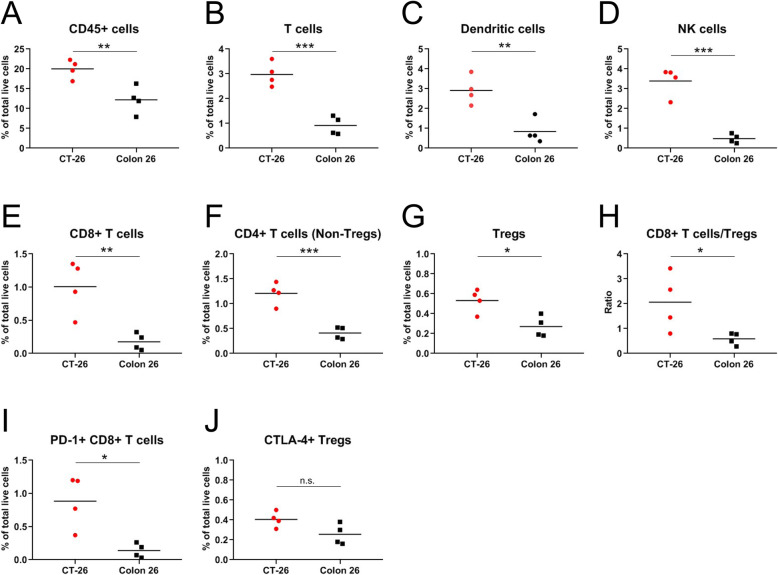
CT-26 tumors are infiltrated with more anti-tumor immune cells than Colon 26 tumors. BALB/c mice were inoculated with 3 × 10^5^ CT-26 or Colon 26 cells. When the tumor volume reached approximately 100 mm^3^, tumor tissues were harvested, dissociated into single cells and analyzed by flow cytometry. All data are represented as percent of total live cells, which include both tumor cells and immune cells. Quantification of (**A**) CD45+ cells, (**B**) T cells (CD45+ CD3+ CD11b-), (**C**) dendritic cells (CD45+ CD11b + CD11c + MHCII+), (**D**) NK cells (CD45+ CD3- CD11b- CD335+), (**E**) CD8+ T cells (CD45+ CD3+ CD11b- CD4- CD8+), (**F**) CD4+ T cells (Non-Tregs, CD45+ CD3+ CD11b- CD4+ CD8-Foxp3-), (**G**) Tregs (CD45+ CD3+ CD11b- CD4+ CD8- CD25+ Foxp3+), (**H**) ratio of CD8+ T cells to Tregs, (**I**) PD-1+ CD8+ T cells and (**J**) CTLA-4+ Tregs. Mean of each immune population are indicated as bars. Statistical significance (t-test) between groups: * 0.01 < *p* < 0.05, ** 0.001 < *p* < 0.01, *** *p* < 0.001. n.s., nonsignificant

The presence of pre-existing lymphocytes and myeloid cells within the tumor is known to influence response to immune checkpoint inhibitors [3]. To evaluate the composition of tumor-infiltrating leukocytes in CT-26 and Colon 26 tumor tissues, baseline tumor tissues from tumor-bearing mice were harvested, dissociated into single cell suspension and immune-profiling evaluated by flow cytometry (Additional file: Fig. S2). Immune infiltrates detected with the pan-leukocyte marker CD45 were present in both CT-26 and Colon 26 tumor tissues, but the frequency of CD45+ cells within live cells are higher in CT-26 than in Colon 26 tumor tissues (Fig. 2A). Leucocyte subsets were evaluated by using a combination of lineage markers to identify specific immune subsets. CT-26 tumor tissues contained more infiltrates of T cells (CD45+ CD3+ CD11b-; Fig. 2B), dendritic cells (CD45+ CD11b + CD11c + MHCII+; Fig. 2C) and NK cells (CD45+ CD3- CD11b- CD355+; Fig. 2D) than Colon 26 tumor tissues. There was no significant difference of frequencies of tumor-B cells (CD45+ CD3- CD11b- CD355- CD19+), Myeloid cells (CD45+ CD3- CD11b+) and Macrophages (CD45+ CD11b + CD11c- Ly6G- Ly6C-F4/80+) between CT-26 and Colon 26 tumor tissues (Additional file: Fig. S3). Next, to reveal tumor infiltrating T cell subsets, we examined surface markers of T cell subsets. The frequencies of CD8+ T cells (CD45+ CD3+ CD11b- CD4- CD8+) and Foxp3-CD4+ T cells (CD45+ CD3+ CD11b- CD4+ CD8- Foxp3-) in CT-26 tumor tissues were significantly higher than those in Colon 26 tumor tissues (Fig. 2E and F). Though the frequency of Tregs (CD45+ CD3+ CD11b- CD4+ CD8- CD25+ Foxp3+) was also higher in CT-26 tumor tissues, (Fig. 2G), the ratio of CD8 to Treg in CT-26 tumor tissues was significantly higher than that in Colon 26 tumor tissues (Fig. 2H). Our data showed that CT-26 tumor tissues exhibited immune-inflamed phenotype characterized by more infiltration with anti-tumor immune cells at baseline than Colon 26. In contrast to tumor tissues, there were no significant differences of frequencies of T cells, NK cells and dendritic cells and CD8 to Treg ratio in the peripheral blood between CT-26 and Colon 26 tumor bearing mice (Additional file: Fig. S4).

In human melanoma patients, anti-PD-1 therapeutic responses were reported to be associated with the presence of PD-1 expressing CD8+ T cells at the tumor margin, co-localized with PD-L1 expressing tumor cells before therapy [21], and therefore PD-1+ CD8+ T cells can be one of the targeted cells by anti-PD-1 mAb. We hypothesized that anti-PD-1 mediated tumor growth inhibition in CT-26 model was due to enhanced frequency of PD-1-expresing CD8 T cells within the tumor. As shown in Fig. 2I, CT-26 tumors at baseline have higher frequency of PD-1 + CD8+ T cells as compared to Colon 26 baseline tumors. Correlating with the sensitivity of both models to anti-CTLA-4 treatment, no notable differences were observed in the frequencies of CTLA-4 expressing Tregs between two tumor types (Fig. 2J). Taken together, these data suggest that the tumor plays an important role in shaping the immune contexture of the TME which may contribute to the sensitivity to ICB therapies.

### Neither TMB nor the number of indels predicts the anti-PD-1 antibody response

**Fig. 3:**
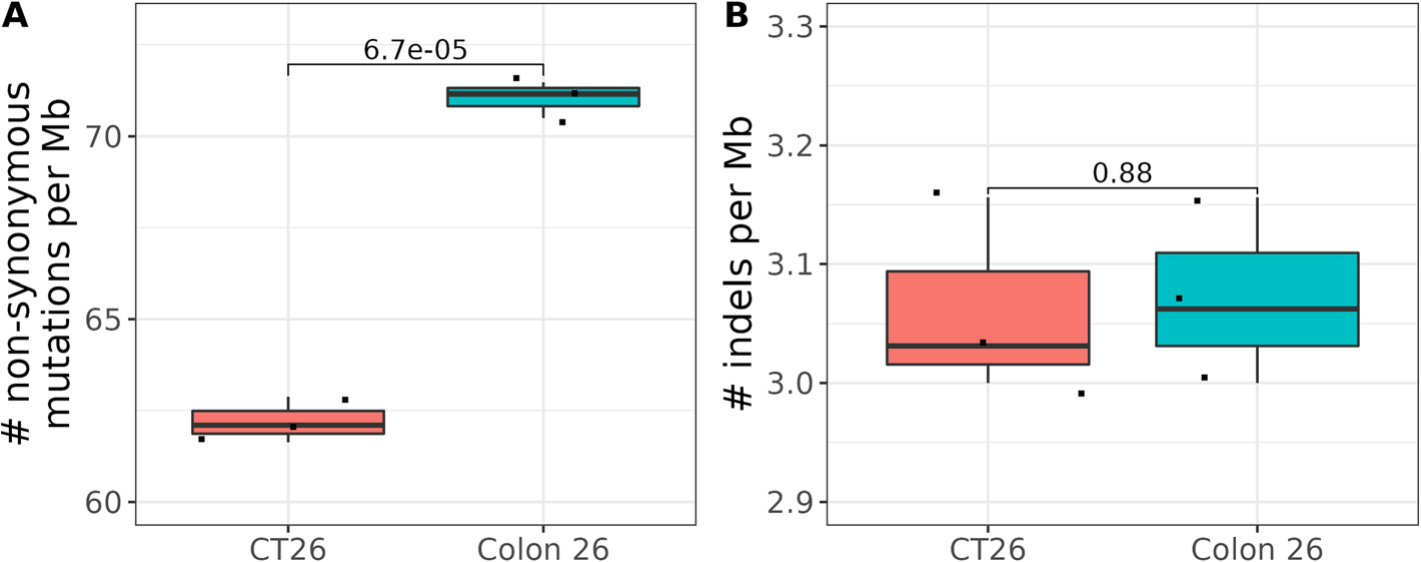
Mutations detected in CT-26 and Colon 26 in cell line samples. **A**. Tumor mutational burden (numbers of non-synonymous mutations per Mb). **B**. Number of indelsper Mb

One of the tumor intrinsic factors that is positively correlated with ICB response is the TMB in multiple cancer types [5], such as melanoma [22, 23], non-small-cell lung carcinoma [24], urothelial carcinoma [6]. To test if TMB can explain the different anti-PD-1 sensitivity of CT-26 and Colon 26, we sequenced their exomes and compared TMB in the cell lines (Fig. 3A, Additional file: Table S1), which revealed that Colon 26, rather CT-26, has higher TMB. Thus, for these two tumor models, it is unlikely that TMB is the critical factor in the observed differential anti-PD-1 sensitivity. Another potential predictive tumor biomarker for ICB response is the number of indels [3], since indels are more likely to produce neoantigens that can be readily detected by the immune system [25]. We further examined the number of indels in these samples and found no significant difference between CT-26 and Colon 26 (Fig. 3B). In summary, in the context of CT-26 and Colon 26, neither TMB nor the number of indels predicts the anti-PD-1 antibody response.

### Wnt pathway is highlighted as one of the most distinct features between CT-26 and Colon 26

To determine the transcriptomic differences between CT-26 and Colon 26, we sequenced RNA from these cell lines as well as tumors isolated ex vivo from tumor bearing animals at sizes 100, 400 and 800 mm^3^. Transcriptome profiles of cell lines and tumor tissues were different, independent of tumor type, likely because the tumor tissue was composed by not only tumor cells but also immune cells and stromal cells. (Additional file: Fig. S5). Thus, we focus on cell line-cell line and tumor-tumor comparisons.

**Fig. 4:**
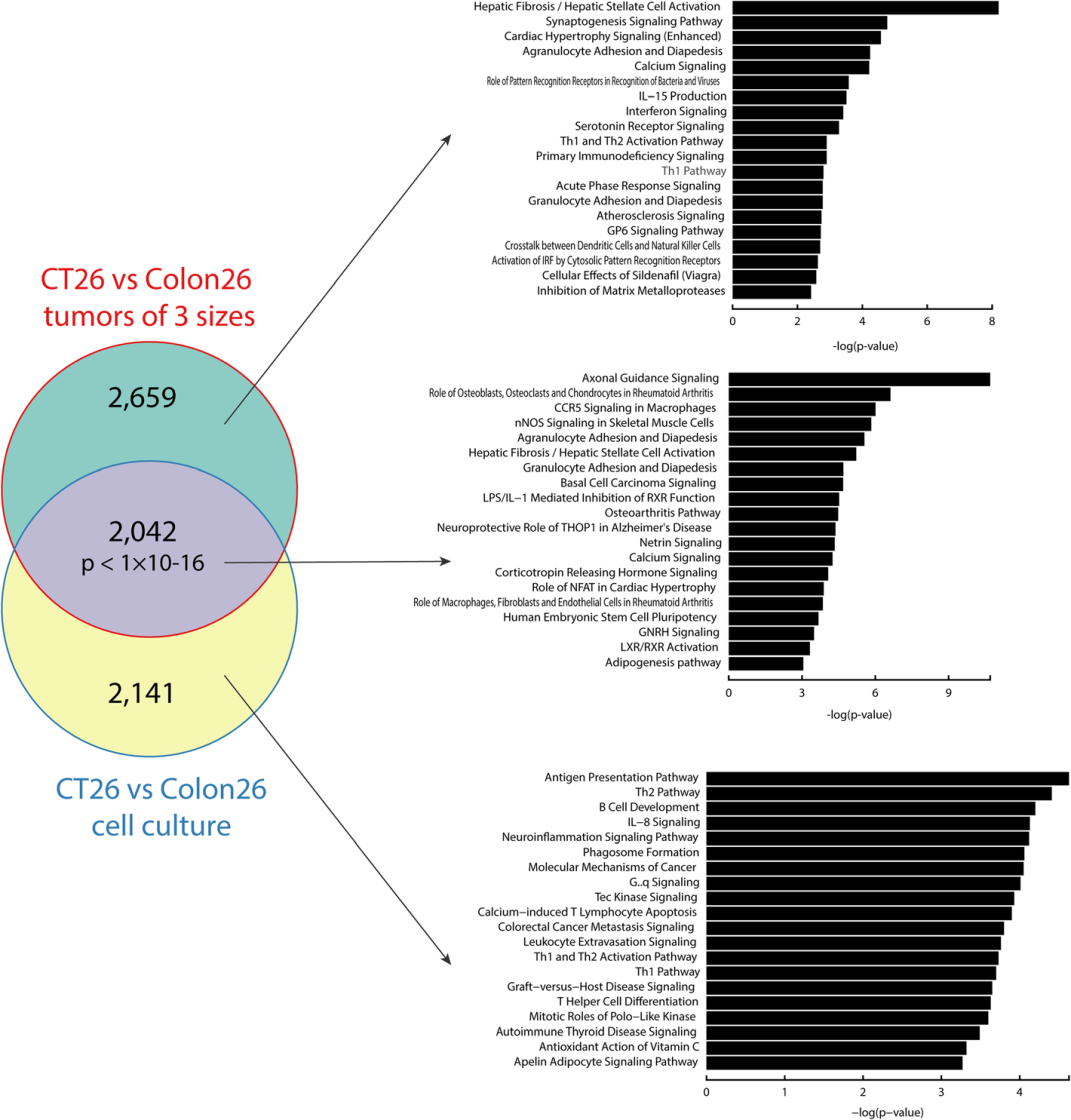
Canonical pathway analysis of differentially expressed genes between CT-26 vs. Colon 26 from tumor tissues of different sizes and cell lines. CT-26 and Colon 26 tumors were compared at 3 different sizes to derive 3 sets of DE genes and the union of the 3 sets contained 4701 DE genes. Comparing CT-26 and Colon 26 cell lines revealed 4183 DE genes. A significant number of DE genes (2042, *p* < 1 × 10^− 6^, hypergeometric test) are shared between the tumor-tumor and cell line-cell line comparisons

Next, we sought to determine which differentially expressed (DE) genes stem from cell lines and which DE genes appear after tumor inoculation (Fig. 4). Comparing CT-26 and Colon 26 at 3 different sizes revealed 3 sets of DE genes, the union of which contained 4701 DE genes. On the other hand, comparingCT-26 and Colon 26 cell lines revealed 4183 DE genes. While 2141 or 2659 DE genes are observed as cell-linespecific or tumor tissue-specific DE genes respectively, a significant number of DE genes (2042) are shared between the tumor-tumor and cell line-cell line comparisons (Fig. 4), suggesting this set of shared DE genes observed in tumors are from tumor cells themselves.

**Fig. 5:**
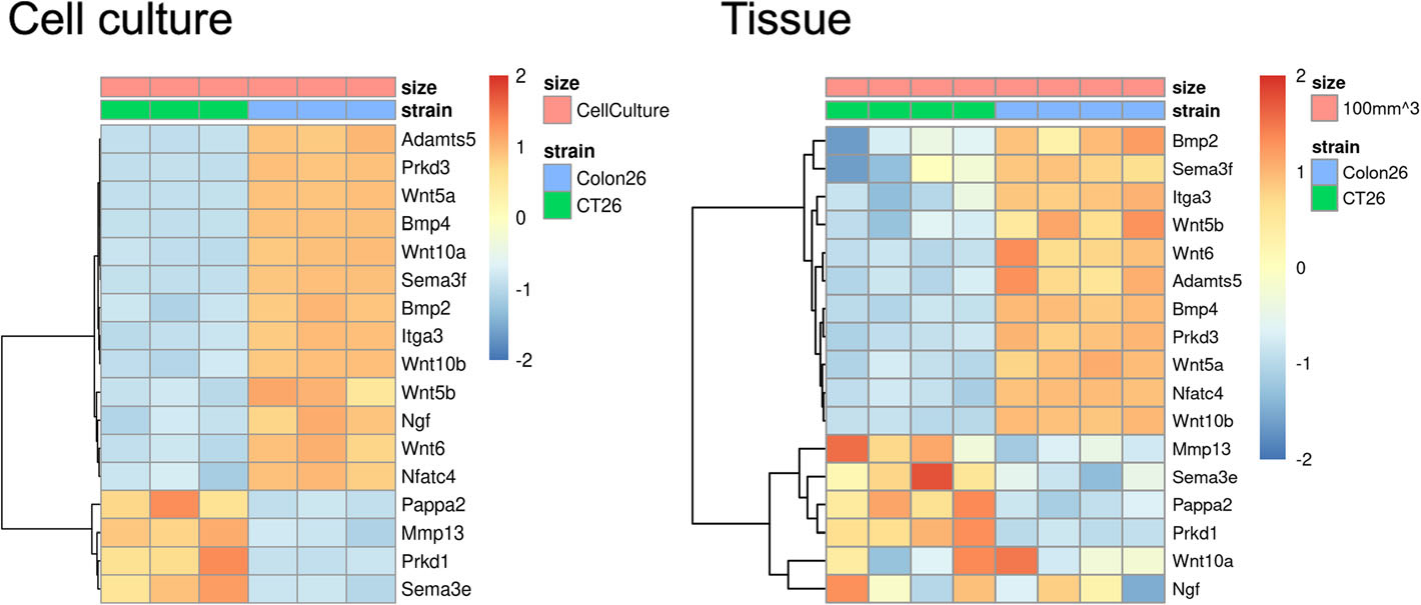
Wnt ligand and Wnt-regulated gene expression. Cell line comparison and comparison of tumors at 100 mm^3^

To understand biological meaning of the DE genes, we performed pathway enrichment analysis of cell line and tumor tissues DE genes. We found that some pathways—such as axonal guidance signaling and hepatic fibrosis—are enriched, regardless of the tumor size (Additional file: Fig. S6). Such pathways are also often enriched in the CT-26-Colon 26 cell line comparison, indicating that tumors recapitulate some biological themes from the cell line. We also performed the pathway analysis of the shared DE genes (2042) and cell-line specific (2141) or tumor tissue specific DE genes (2659) (Fig. 4). Consistent with above pathway analysis result, axon guidance signaling is the most significant pathway in the shared DE genes. Wnts are conserved guidance molecules for a large numbers of central nervous system axons and Wnt signaling plays an important role in axon guidance [26–28]. Almost all colorectal cancers demonstrate hyperactivation of the Wnt pathway, which in many cases is believed to be the initiating and driving event [29]. We found that this pathway contains major Wnt ligands (Wnt5a, Wnt5b, Wnt6, Wnt10a, and Wnt10b) and the Wnt-upregulated genes (Adamts5, Bmp2, Bmp4, Itga3, Nfatc4, Ngf, Prkd3, and Sema3f) or downregulated genes (Mmp13, Pappa2, Prkd1, and Sema3e) (Fig. 5) [30–33]. Almost all of these Wnt ligands and Wnt-upregulated genes were upregulated in Colon 26 and all downregulated genes were downregulated in Colon 26 (Fig. 5), showing that Wnt activity in the tumor tissue was higher in Colon 26 than CT-26 and the different Wnt pathway activities play a key role in delineating the two cell lines. In addition, Wnt/β-catenin signaling was statistically significantly enriched in the shared DE genes (*p* = 0.026). Previously, the Wnt pathway has been reported to play a role in responses to checkpoint blockades [34]. With these observations from the comparison of CT-26 and Colon 26, we hypothesize that Wnt pathway signaling may explain the differential anti-PD-1 antibody response in CT-26 and Colon 26.

## Discussion

In this study, we identified CT-26 and Colon 26 as different sensitive tumor models to anti-PD-1 therapy by in vivo screening and performed deep tumor-immune characterizations of the two syngeneic models to understand determinants driving anti-tumor activities of ICB. CT-26 is a murine colorectal carcinoma derived from BALB/c mice, and the syngeneic tumor model is one of the most commonly used murine solid tumor models and is categorized as a highly immunogenic tumor model [35]. Colon 26 is also a murine colorectal carcinoma derived from BALB/c mice, but the syngeneic tumor model has not been reported as frequently as CT-26 model. For Colon 26 tumor model, its sensitivity to immunotherapies, immune contexture of the TME and the differences from other commonly used murine tumor models has not been fully documented. We evaluated anti-tumor efficacies of anti-mPD-1 and anti-mCTLA-4 mAbs in the CT-26 and Colon 26 tumor bearing mice. Although the same cohort BALB/c mice were used for the efficacy study, sensitivities to anti-PD-1 were different between the models, suggesting that host genetics and microbiota before tumor inoculation do not affect the sensitivities. Multiple studies in a variety of tumor types have found a positive correlation between tumoral PD-L1 expression and ICB response or overall survival, while others have detected no association [3]. PD-1 blockade with pembrolizumab is approved by FDA for non-small-cell lung carcinoma patients whose tumors are positive for PD-L1 expression [36]. However, it’s an imperfect biomarker since in some tumors, such as renal cell carcinoma and first-line bladder cancer [37], there appears to be no correlation between PD-L1 expression and the likelihood of clinical response. In this study, we also performed immunohistochemistry analysis to determine PD-L1 expression level in the tumor (Additional file: Fig. S7), and there was no statistically difference of the expression between CT-26 and Colon 26 tumor tissues. This suggested that tumoral PD-L1 expression may not explain the different sensitivity to anti-mPD-1 mAbs.

In contrast to anti-mPD-1, sensitivities to anti-mCTLA-4 were similar between the models. CTLA-4-blocking antibodies augment the binding of CD80/86 to CD28 rather than to CTLA-4 which triggers T-cell survival and expansion [38]. It also selectively depletes intra-tumoral CTLA-4 expressing Tregs via FcγR dependent mechanisms [39, 40]. At baseline, there was no notable difference of CTLA-4 expressing Tregs in the tumor-bearing mice between the two models. There was also similar expression of Cd80, Cd86, Fcgr1, Fcgr3 and Fcgr4 in the tumor tissues (data not shown). These data may reflect similar sensitivities to anti-mCTLA-4 mAbs between CT-26 and Colon 26 models.

We found that baseline immune population in the tumor was different between CT-26 and Colon 26. Compared to Colon 26 tumors, CT-26 tumors were more infiltrated by not only CD45+ immune cells but also anti-tumor immune cells including CD8 + T cells, NK cells and DCs. Chemokines can determine the distribution of immune cells in the tumor. CXC-chemokine receptor 3 (CXCR3) and its ligands CXC-chemokine ligand 10 (CXCL10) and CXCL11 have a key role in driving the trafficking of Th1 cells, CD8+ T cells and NK cells into the TME [41]. Our data showed that gene expression levels of Cxcl10 and Cxcl11 in the 100 mm^3^ CT-26 tumors are higher than Colon 26 (Additional file: Fig. S8). This may reflect more CD8+ T cells and NK cells infiltration to the CT-26 tumor than Colon 26.

Despite growing literature reports supporting the association between mutational burden and immune checkpoint therapy response, tumor mutational burden had poor predictive power to differentiate complete or partial response from progressive disease as a single variable [42]. In this study, Colon 26 has higher TMB than CT-26. Thus, TMB cannot explain the differential anti-mPD-1 sensitivity for our two tumor models. Nevertheless, we cannot ruled out other genetic features beyond mutational burden—such as genetic driver events, intra-tumoral heterogeneity, and mutational signatures—may affect response to immune checkpoint blockade [42].

Colorectal cancer (CRC) is the third most predominant cancer throughout the world [43]. Sonic Hedgehog, Wnt/ß-catenin, TGF-ß/SMAD, EGFR and Notch pathways are the major pathways that could be targeted for CRC therapy [43]. MSI-positive CRC patients have demonstrated positive response with ICB therapy [1]. We examined a few MMR related genes expression and found that MLH1 and MSH6 gene expression were higher in Colon 26 cell and tissue than CT-26, suggesting MMR difference might not explain differential anti-PD1 response. However, further investigation will be needed to clarify the difference of MMR gene expression leads to functional effects on MMR.

Interestingly, our transcriptome analysis highlighted Wnt pathway as one of the most distinct features between CT-26 and Colon 26. Wnt signaling has been shown to play a major role in regulating the immune tolerance against tumors [34]. Excluding immune cell (mainly CTL) infiltration into the TME constitutes a prominent mechanism of Wnt-mediated immuno-evasion in different types of cancer, mainly melanomas [14]. Activation of Wnt/β-catenin pathway in cancer cells inhibits secretion of CCL4 that is required for the recruitment of BATF-3 dependent dendritic cells to the tumor micro-environment, resulting in reduced levels of DC-derived CXCL10 and limited CD8+ CTL infiltration and cross-priming [14]. In consistent with this mechanism, we also observed less infiltration of dendritic cells and CD8+ T cells, as well as low expression of Cxcl10 and Xcr1 (a marker for cross-presenting CD8α + DCs) in Colon 26 tumor tissues (100 mm^3^) than CT-26.It’s been reported that the lack of effector cells into the TME, as typically observed in tumor with active canonical Wnt signaling, is possibly a main cause of primary resistance to cancer immunotherapies with ICB [34]. In mouse melanoma models, tumor-intrinsic active β-catenin signaling results in T-cell exclusion and resistance to combination of anti-PD-L1 and anti-CTLA-4 mAbs [14]. Conversely, RNAi mediated β-catenin inhibition increased T cell infiltration in ICB-refractory syngeneic mouse melanoma tumors [44]. Our study provides additional evidence and supports the potential resistance mechanism in mouse colon models.

With these observation from the comparison of CT-26 and Colon 26, we hypothesize that Wnt pathway signaling may explain the differential anti-PD-1 antibody response in CT-26 and Colon 26. Colon 26 might be resistant to anti-PD-1 therapy, because of high Wnt activity in the tumor tissue and excluded immune cells, while CT-26 might be sensitive to the therapy, because of low Wnt activity and a failure to exclude immune cells. In our study, we did not test the impact of Wnt signal inhibition on sensitivity to anti-PD-1 in Colon 26. In order to validate our Wnt hypothesis, we will need to generate β-catenin knock-out Colon 26 and compare sensitivity to anti-PD-1 treatment in the knock-out and the parental Colon 26 tumor bearing mice or test inhibitors of Wnt ligand secretion, such as LGK-974 [44], by combining with anti-PD-1 treatment in Colon 26 tumor models. Unlike anti-PD-1, there was no notable different sensitivity to anti-CTLA-4. We also need further investigation on whether Wnt pathway in the tumor will specifically affect sensitivity to anti-PD-1 therapy or not.

## Conclusions

We evaluated anti-tumor effect of ICB in CT-26 and Colon 26 tumor bearing mice and found that CT-26 tumor bearing mice were more sensitive to anti-PD1 therapy than Colon 26. We profiled the genomic and genetic expression and cellular phenotype in both syngeneic models and found that CT-26 was a “hot” tumor type with more tumor-infiltrating immune cells than Colon 26 and that axon guidance signaling with Wnt molecules were highlighted in Colon 26 more than CT-26. Based on these observation and previous reports, Wnt pathway may explain different anti-tumor activity between CT-26 and Colon 26 syngeneic tumor models. Our approach to focus on preclinical tumor models with similar genetic background but different sensitivity to anti-PD-1 therapy would contribute to understanding mechanism of anti-PD-1 response more clearly and to generating a novel concept to synergize anti-tumor activity of anti-PD-1and to target anti-PD-1 non-responder patients.

## Supplementary Information

**Additional file 1.**

**Additional file 2.**

**Additional file 3.**

**Additional file 4.**

**Additional file 5.**

**Additional file 6.**

**Additional file 7.**

**Additional file 8.**

**Additional file 9.**

## Data Availability

RNA-seq data is available from Gene Expression Omnibus database (NCBI) with accession number GSE185023 (https://www.ncbi.nlm.nih.gov/geo/query/acc.cgi?acc=GSE185023).WES data is available from Sequence Read Archive (SRA) with accession number PRJNA767812 (https://www.ncbi.nlm.nih.gov/sra/PRJNA767812).

## Abbreviations

BATF-3: Basic leucine zipper transcription factor ATF-like 3
CCL: C-C-ligand
cDNA: complementary deoxyribonucleic acid
CDS: Coding sequence
CRC: Colorectal cancer
CTL: Cytotoxic T lymphocytes
CTLA-4: Cytotoxic T lymphocyte-associated antigen 4
CXCL: CXC-chemokine ligand
CXCR: CXC-chemokine receptor
DCs: Dendritic cells
DE: Differentially expressed
dsDNA: double stranded deoxyribonucleic acid
EGFR: Epidermal growth factor receptor
FBS: Fatal bovine serum
FcγR: Fcγ receptor
FDA: Food and drug administration
IACUC: Institutional Animal Care and Use Committees
ICB: Immune checkpoint blockade
JAK: Janus kinase
mAb: monoclonal antibody
MAPK: Mitogen-activated protein kinase
MHC: Major histocompatibility complex
MMR: Mismatch repair
MSI: Microsatellite instability
NF-κB: nuclear factor-κB
NK cells: Natural killer cells
NSCLC: Non-small cell lung cancer
nsSNVs: non-synonymous single nucleotide variants
PBS: Phosphate-buffered saline
PCR: Polymerase chain reaction
PD-1: Programmed cell death protein 1
PD-L1: Programmed death-ligand 1
RNA: Ribonucleic acid
SEM: Standard error of the mean
SNPs: Single nucleotide polymorphisms
STAT3: Signal transducer and activator of transcription 3
Th1: T helper 1
TME: Tumor-micro-environment
TMB: Tumor mutation burden
Tregs: Regulatory T cells
