# Supplementary material for: Tumor-immune profiling of CT-26 and Colon 26 syngeneic mouse models reveals mechanism of anti-PD-1 response
Source: BMC Cancer. 2021 Nov 13;21:1222. doi: 10.1186/s12885-021-08974-3 (PMC8590766; doi:10.1186/s12885-021-08974-3)
Supplement: Supplementary file 1 — Additional file 1. [file 12885_2021_8974_MOESM1_ESM.pdf]

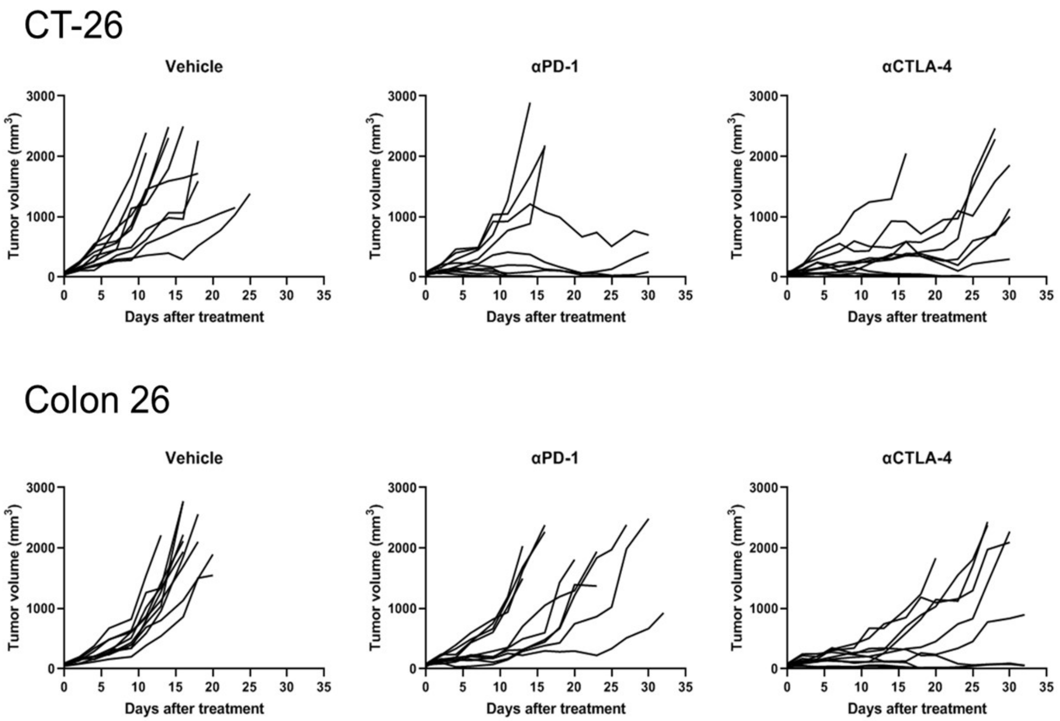

Figure S1. Different sensitivities to anti-PD-1 and anti-CTLA-4 checkpoint blockades in CT-26 and Colon 26 tumor-bearing mice

Spider plots of CT-26 and Colon 26 tumor volume for individual mice treated with vehicle (PBS), anti-PD-1 mAbs and anti-CTLA-4 mAbs
