# Supplementary material for: Tumor-immune profiling of CT-26 and Colon 26 syngeneic mouse models reveals mechanism of anti-PD-1 response
Source: BMC Cancer. 2021 Nov 13;21:1222. doi: 10.1186/s12885-021-08974-3 (PMC8590766; doi:10.1186/s12885-021-08974-3)
Supplement: Supplementary file 2 — Additional file 2. [file 12885_2021_8974_MOESM2_ESM.pdf]

A

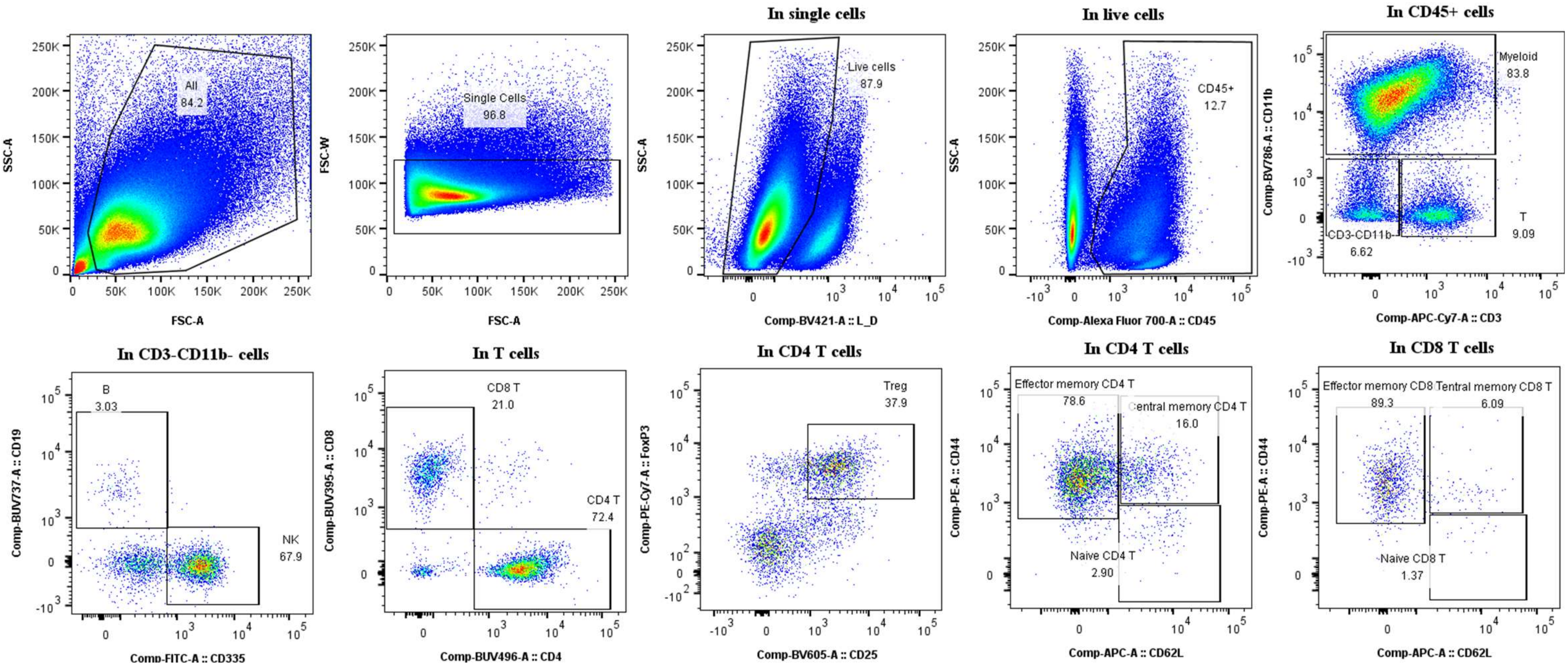

All
84.2
SSC-A
FSC-A
Single Cells
96.8
FSC-W
In single cells
Live cells
87.9
Comp-BV421-A :: L_D
In live cells
CD45+
12.7
Comp-Alexa Fluor 700-A :: CD45
In CD45+ cells
Myeloid
83.8
T
9.09
CD3-CD11b-
6.62
Comp-BV786-A :: CD11b
Comp-APC-Cy7-A :: CD3
In CD3-CD11b- cells
B
3.03
NK
67.9
Comp-BUV737-A :: CD19
Comp-FITC-A :: CD335
In T cells
CD8 T
21.0
CD4 T
72.4
Comp-BUV395-A :: CD8
Comp-BUV496-A :: CD4
In CD4 T cells
Treg
37.9
Comp-PE-Cy7-A :: FoxP3
Comp-BV605-A :: CD25
In CD4 T cells
Effector memory CD4 T
78.6
Central memory CD4 T
16.0
Naive CD4 T
2.90
Comp-PE-A :: CD44
Comp-APC-A :: CD62L
In CD8 T cells
Effector memory CD8
89.3
Tentral memory CD8 T
6.09
Naive CD8 T
1.37
Comp-PE-A :: CD44
Comp-APC-A :: CD62L

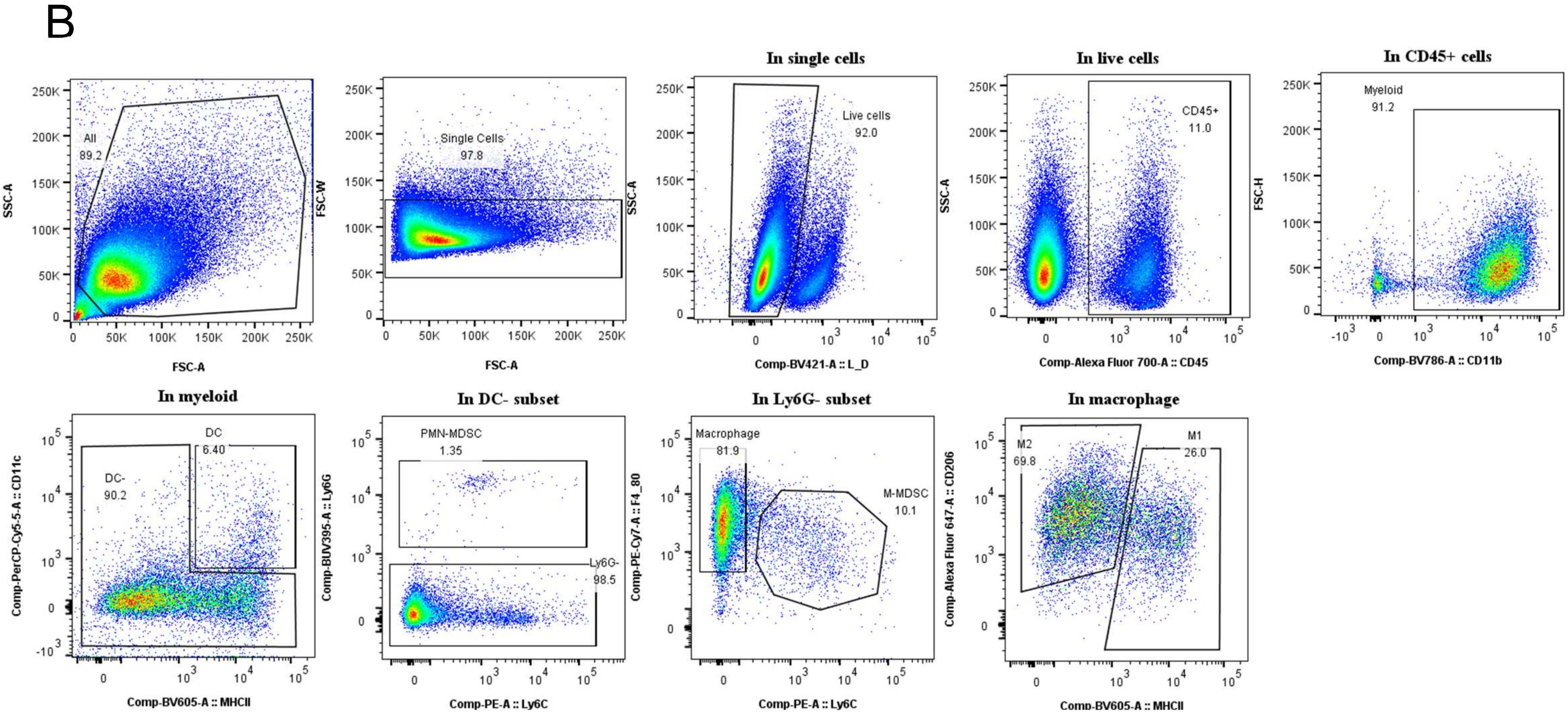

Figure S2. Flow cytometry gating strategy for tumor-infiltrating immune cells analysis

(A) Panel for lymphocytes and (B) panel for myeloid cells
