# Supplementary material for: Tumor-immune profiling of CT-26 and Colon 26 syngeneic mouse models reveals mechanism of anti-PD-1 response
Source: BMC Cancer. 2021 Nov 13;21:1222. doi: 10.1186/s12885-021-08974-3 (PMC8590766; doi:10.1186/s12885-021-08974-3)
Supplement: Supplementary file 3 — Additional file 3. [file 12885_2021_8974_MOESM3_ESM.pdf]

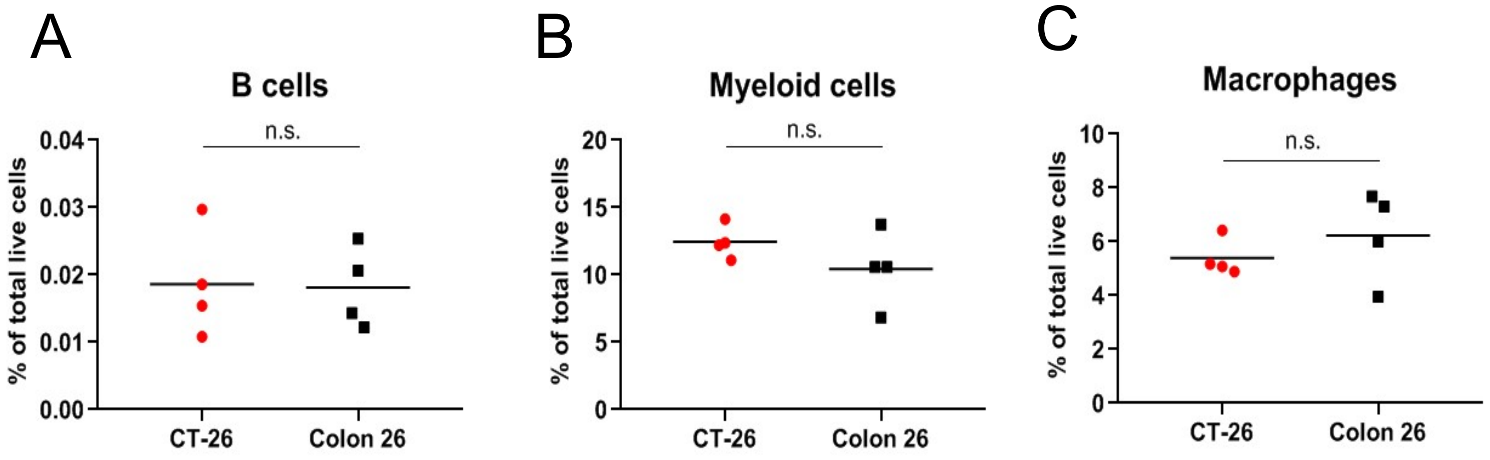

Figure S3. No difference of frequencies of B cells, myeloid cells and macrophages between CT-26 and Colon 26 tumor tissues.

BALB/c mice were inoculated with 3 x 10^5 CT-26 or Colon 26 cells. When the tumor volume reached approximately 100 mm^3, tumor tissues were are harvested, dissociated into single cells and analyzed by flow cytometry. All data are represented as percent of total live cells, which include both tumor cells and immune cells. Quantification of (A) B cells (CD45+ CD3- CD11b- CD335- CD19+), (B) Myeloid cells (CD45+ CD11b+) and (C) Macrophage (CD45+ CD11b+ CD11c- Ly6G- Ly6C- F4/80+). Means of each immune population are indicated as bars. n.s., nonsignificant
