# Supplementary material for: Tumor-immune profiling of CT-26 and Colon 26 syngeneic mouse models reveals mechanism of anti-PD-1 response
Source: BMC Cancer. 2021 Nov 13;21:1222. doi: 10.1186/s12885-021-08974-3 (PMC8590766; doi:10.1186/s12885-021-08974-3)
Supplement: Supplementary file 4 — Additional file 4. [file 12885_2021_8974_MOESM4_ESM.pdf]

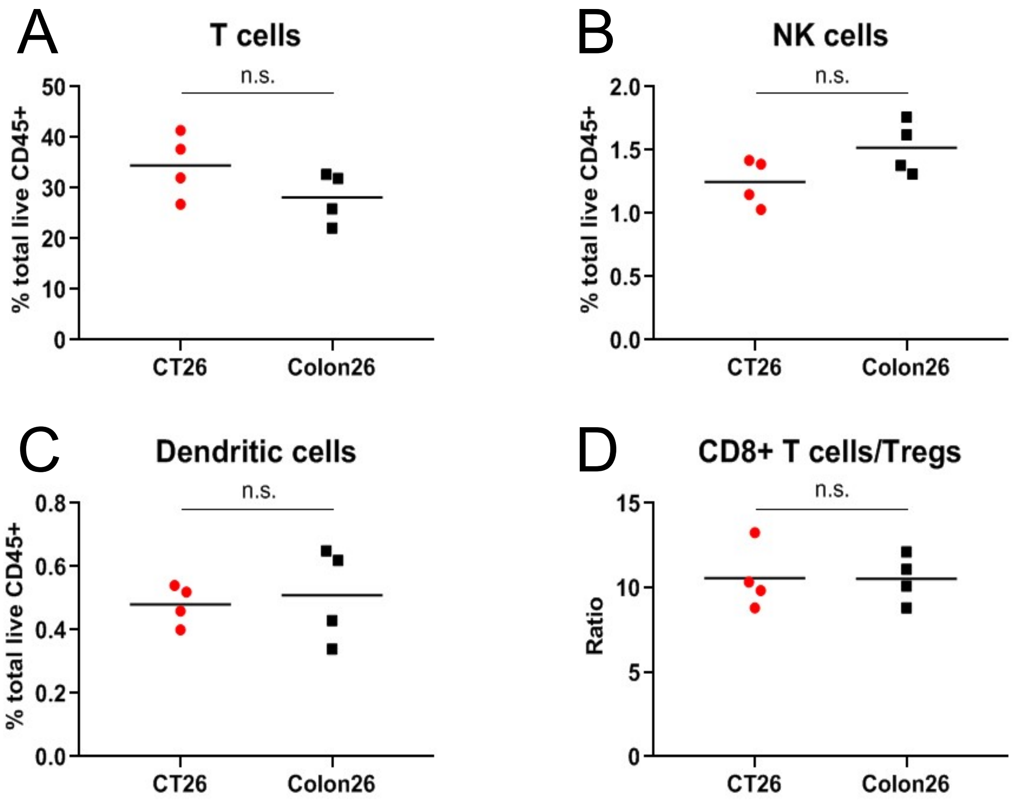

Figure S4. No difference of frequencies of T cells, NK cells and dendritic cells and ratio of CD8+ T cells to Tregs in the peripheral blood between CT-26 and Colon 26 tumor bearing mice.

BALB/c mice were inoculated with 3 x 10^5 CT-26 or Colon 26 cells. When the tumor volume reached approximately 100 mm^3, peripheral blood was collected, lysed and analyzed by flow cytometry. All data are represented as percent of total live CD45+ cells. Quantification of (A) T cells, (B) NK cells, (C) dendritic cells and (D) ratio of CD8+ T cells to Tregs. Means of each immune population are indicated as bars. n.s., nonsignificant
