# Supplementary material for: Tumor-immune profiling of CT-26 and Colon 26 syngeneic mouse models reveals mechanism of anti-PD-1 response
Source: BMC Cancer. 2021 Nov 13;21:1222. doi: 10.1186/s12885-021-08974-3 (PMC8590766; doi:10.1186/s12885-021-08974-3)
Supplement: Supplementary file 5 — Additional file 5. [file 12885_2021_8974_MOESM5_ESM.pdf]

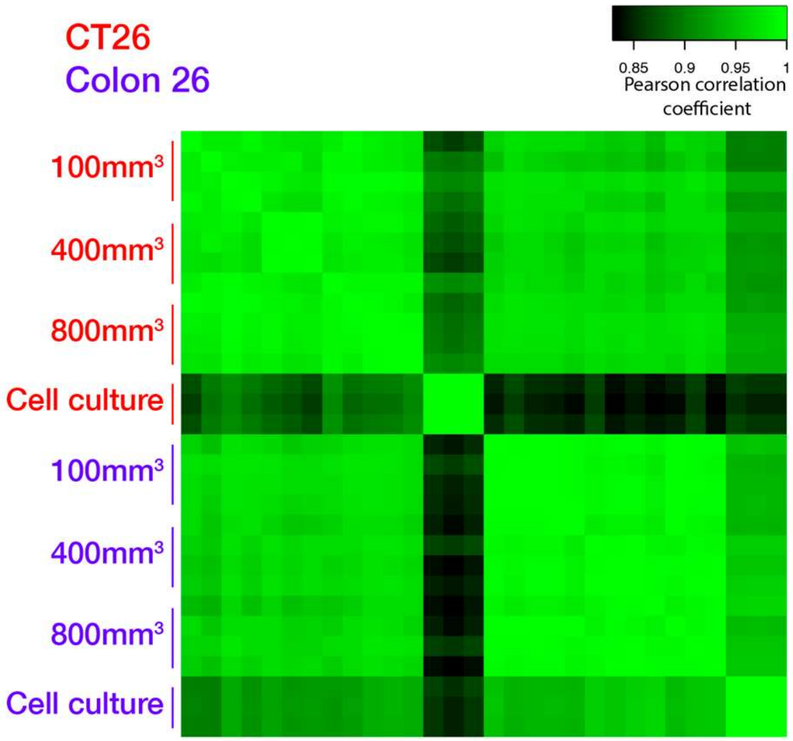

Figure S5. Correlations of gene expression levels in all RNA-seq samples.

Row and columns are in the same order.
