# Supplementary material for: Tumor-immune profiling of CT-26 and Colon 26 syngeneic mouse models reveals mechanism of anti-PD-1 response
Source: BMC Cancer. 2021 Nov 13;21:1222. doi: 10.1186/s12885-021-08974-3 (PMC8590766; doi:10.1186/s12885-021-08974-3)
Supplement: Supplementary file 6 — Additional file 6. [file 12885_2021_8974_MOESM6_ESM.pdf]

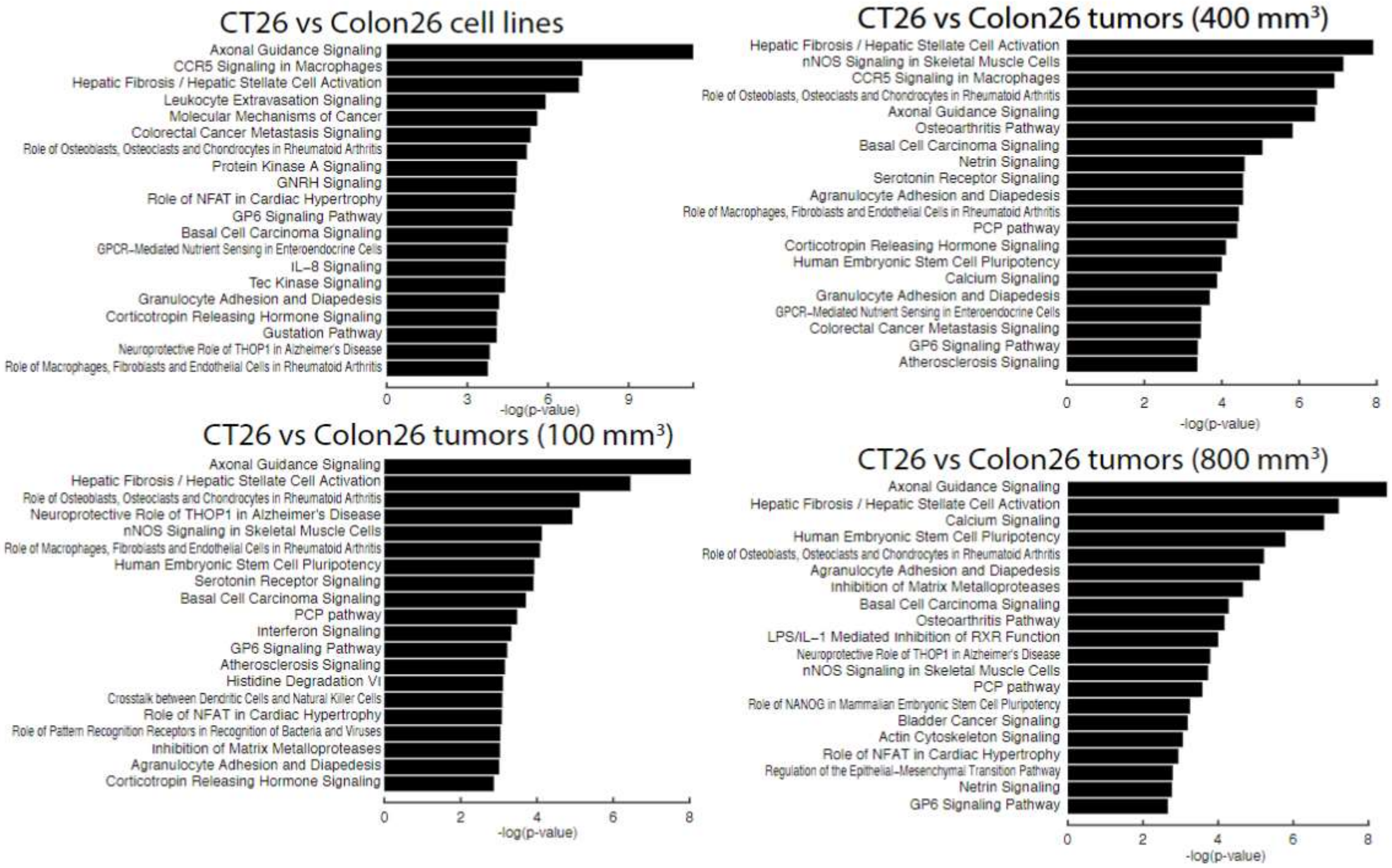

Figure S6. Canonical pathway analysis of differentially expressed genes categorized according to their sources.
