# Supplementary material for: Tumor-immune profiling of CT-26 and Colon 26 syngeneic mouse models reveals mechanism of anti-PD-1 response
Source: BMC Cancer. 2021 Nov 13;21:1222. doi: 10.1186/s12885-021-08974-3 (PMC8590766; doi:10.1186/s12885-021-08974-3)
Supplement: Supplementary file 7 — Additional file 7. [file 12885_2021_8974_MOESM7_ESM.pdf]

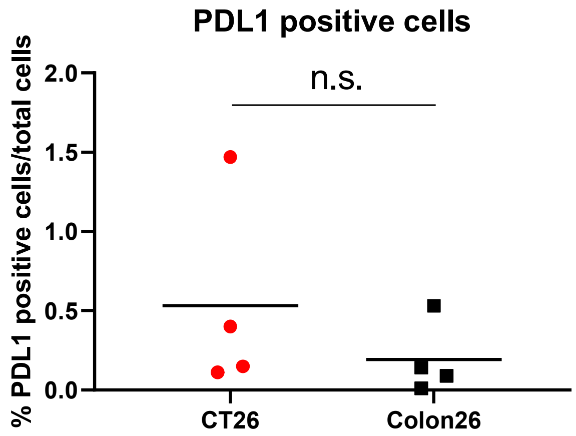

Figure S7. No difference of PDL1 positive cells between CT-26 and Colon 26 tumor tissues.

BALB/c mice were inoculated with 3 x 10^5 CT-26 or Colon 26 cells. When the tumor volume reached approximately 100 mm^3, tumor tissues were harvested. Immunohistochemistry analysis was performed on fixed and paraffin embedded tumor samples. Means are indicated as bars. n.s., nonsignificant
